# Assessment fluid responsiveness in septic shock patients: a comparison of automated pulse pressure variation and manually calculated pulse pressure variation

**DOI:** 10.1186/cc10841

**Published:** 2012-03-20

**Authors:** S Panyawatanaporn, B Khwannimit

**Affiliations:** 1 Prince of Songkla University, Hat Yai, Thailand

## Introduction

Pulse pressure variation (PPV) is an accurate predictor of fluid responsiveness in mechanically ventilated patients. The aim of this study was the assessment and comparison of the ability of automated PPV, when measured by an IntelliVue MP 70 monitor, and manually calculated PPV to predict fluid responsiveness in mechanically ventilated septic shock patients.

## Methods

We conducted a prospective study on 36 septic shock patients. Automated and manually calculated PPV and other hemodynamic data were recorded before and after fluid administration of 500 ml of 6% hydroxyethyl starch (130/0.4) over 30 minutes. Responders were defined as patients with an increase in their cardiac index >15% after fluid loading.

## Results

The agreement (mean bias ± SD) between automated and manually calculated PPV was 4.03 ± 7.37%. The baseline automated PPV correlated with the baseline manually calculated PPV (*r *= 0.79, *P *< 0.01). Twenty-three (63.9%) patients were classified as fluid responders. Automated PPV and manually calculated PPV were significantly higher in responders than in nonresponders (16.0 ± 4.5% vs. 7.2 ± 2.0% and 11.1 ± 5.6 vs. 4.6 ± 2.8%, respectively; *P *< 0.001 for both). The area under the receiver operating characteristic curves of automated PPV was significantly greater than the manually calculated PPV (0.982 vs. 0.87, respectively; *P *= 0.04). The optimal threshold values for predicting fluid responsiveness were 11% for automated PPV (sensitivity 91.3%, specificity 92.3%) and 13% for manually calculated PPV (sensitivity 73.9%, specificity 84.6%).

## Conclusion

Our results indicate that the automated PPV, obtained by the IntelliVue MP 70 monitor, and manually calculated PPV, showed comparable performance for predicting fluid responsiveness in passively ventilated septic shock patients.

